# Evaluation of Unintended Consequences of COVID-19 Pandemic Restrictions and Obesity Prevalence Among Youths

**DOI:** 10.1001/jamanetworkopen.2023.23596

**Published:** 2023-07-14

**Authors:** Stella T. Lartey, Wasantha P. Jayawardene, Stephanie L. Dickinson, Xiwei Chen, Nana Gletsu-Miller, David K. Lohrmann

**Affiliations:** 1 St Jude Children’s Research Hospital, Memphis, Tennessee; 2 School of Human Sciences, Southern Illinois University, Carbondale; 3 Biostatistics Consulting Center, Indiana University School of Public Health, Bloomington; 4 Department of Applied Health Science, Indiana University School of Public Health, Bloomington

## Abstract

This cohort study examines the association between COVID-19 pandemic restrictions and obesity prevalence among youths aged 2 to 19 years in Monroe County, Indiana.

## Introduction

Childhood obesity is linked to adulthood obesity and to childhood and adulthood noncommunicable diseases.^1,2^ Pandemic restrictions imposed in early 2020 contributed to decreased physical activity, reduced access to healthy foods, and increased stress, screen time, and consumption of processed foods and sugary drinks among youths.^3,4,5^ Recent studies provide limited insight on longer-term associations of COVID-19 restrictions with childhood obesity prevalence. Thus, this study aimed to evaluate whether pandemic-related body mass index (BMI) changes, if any, among children persisted after restriction removal and to identify associated factors.

## Methods

This cohort study was conducted using data for Monroe County, Indiana, which encompasses 2 municipalities, 2 public school systems, and extensive agrarian and forested areas and had a 2020 population of approximately 148 000 (including 22 000 youths aged 2-19 years).^6^ Weight and height measurements were obtained from electronic health record data for children aged 2 to 19 years in repeated cross-sections (2016-2021; annual average n = 14 595) and for longitudinal cohorts aged 5 to 11 years in 2019 (cohort 1, 2017-2019; n = 8647) and 2021 (cohort 2, 2019-2021; n = 7816). The BMI categories (underweight, healthy weight, overweight, obesity, and severe obesity) were based on the US Centers for Disease Control and Prevention growth charts for age and sex^1,2^ (details in the Figure). Binomial logistic regression was used to establish trends in BMI prevalence and associated factors, including age, sex, residence (zip code group), and health insurance type (race and ethnicity data were not available). Pearson χ^2^ was used to compare transition trends (upward, downward, or stable) between BMI categories for both cohorts by longitudinally tracking individuals with complete responses for 3 consecutive years. The Indiana University Health Bloomington Hospital and Indiana University Bloomington institutional review boards approved this study and waived informed consent because deidentified patient data were used. The study followed the STROBE reporting guideline.

**Figure.**
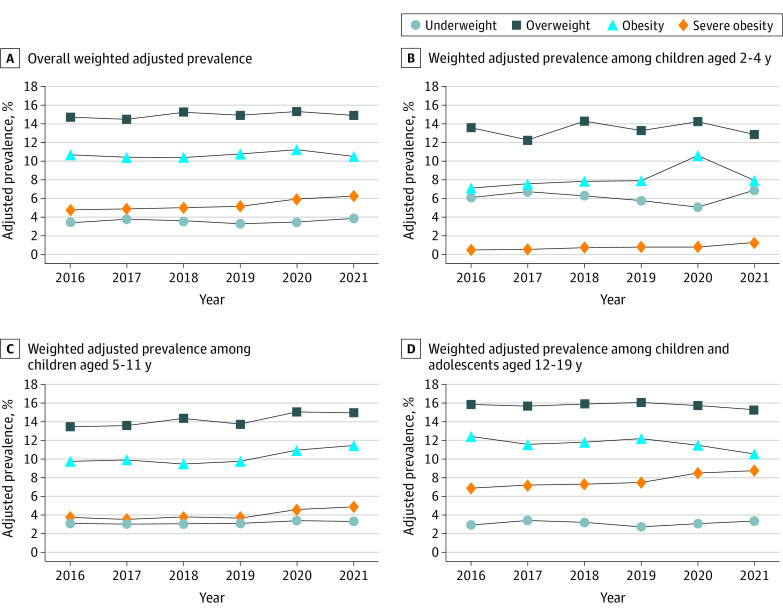
Adjusted Prevalence of Body Mass Index (BMI) Categories for Children Aged 2 to 19 Years, 2016 to 2021 Data are from the electronic health records of children aged 2 to 19 years. A to D, Prevalence estimates are weighted and presented overall (A) and for children aged 2 to 4 years (B), 5 to 11 years (C), and 12 to 19 years (D). The BMI categories were defined based on the US Centers for Disease Control and Prevention BMI-for-age growth chart as underweight (<5th percentile), healthy (5th-<85th percentile), overweight (85th-<95th percentile), obesity (≥95th percentile), and severe obesity (≥120% of 95th percentile).

Data analysis was performed between April 2021 and April 2022. Results are presented as percentages or odds ratios (ORs) with 95% CIs. *P* < .05 (2-tailed) was statistically significant. All analyses were performed in Stata, version 17 (StataCorp).

## Results

This study included 27 093 participants (13 497 girls [49.8%] and 13 596 boys [50.2%]; mean [SD] age, 9.8 [5.3] years). Overall severe obesity prevalence was higher in 2020 (5.9% [5.5%-6.4%]; *P* = .009) and 2021 (6.3% [5.8%-6.7%]; *P* < .001) compared with 2019 (5.1% [4.6%-5.5%]) (Figure and Table).

**Table.** Estimated Yearly Overall and Age-Specific Prevalence of Body Mass Index (BMI) Categories From Binomial Logistic Models^a^

Year	BMI category														
	Underweight (<5th percentile)			Healthy weight (5th-<85th percentile)			Overweight (85th-<95th percentile)			Obesity (≥95th percentile)			Severe obesity (≥120% of 95th percentile)		
	Adjusted prevalence, % (95% CI)	OR (95% CI)	*P* value	Adjusted prevalence, % (95% CI)	OR (95% CI)	*P* value	Adjusted prevalence, % (95% CI)	OR (95% CI)	*P* value	Adjusted prevalence, % (95% CI)	OR (95% CI)	*P* value	Adjusted prevalence, % (95% CI)	OR (95% CI)	*P* value
Total sample															
2016	3.5 (3.1-3.8)	1.04 (0.90-1.21)	.56	66.2 (65.3-67.2)	1.02 (0.96-1.08)	.57	14.8 (14.1-15.5)	0.99 (0.91-1.07)	.78	10.7 (10.9-11.4)	1.00 (0.91-1.09)	.94	4.8 (4.4-5.3)	0.93 (0.82-1.07)	.32
2017	3.8 (3.4-4.2)	1.15 (0.99-1.33)	.07	66.4 (65.4-67.3)	1.03 (0.97-1.09)	.41	14.5 (13.8-15.3)	0.97 (0.89-1.05)	.43	10.4 (9.8-11.0)	0.96 (0.88-1.05)	.41	4.9 (4.4-5.3)	0.95 (0.83-1.09)	.47
2018	3.6 (3.3-4.0)	1.10 (0.95-1.27)	.20	65.7 (64.8-66.7)	0.99 (0.94-1.05)	.84	15.2 (14.5-15.9)	0.97 (0.89-1.05)	.60	10.4 (9.8-11.0)	0.96 (0.88-1.05)	.41	5.1 (4.6-5.5)	0.99 (0.87-1.13)	.85
2019	3.3 (3.0-3.7)	1 [Reference]	NA	65.8 (64.9-66.7)	1 [Reference]	NA	14.9 (14.3-15.6)	1 [Reference]	NA	10.8 (10.3-11.3)	1 [Reference]	NA	5.1 (4.7-5.6)	1 [Reference]	NA
2020	3.5 (3.2-3.9)	1.05 (0.91-1.22)	.47	64.0 (63.1-65.0)	0.92 (0.87-0.98)	.005	15.3 (14.6-16.0)	1.02 (0.95-1.10)	.43	11.2 (10.6-11.8)	1.05 (0.96-1.14)	.30	5.9 (5.5-6.4)	1.18 (1.04-1.33)	.009
2021	3.9 (3.5-4.3)	1.17 (1.02-1.35)	.03	64.5 (65.4-63.5)	0.94 (0.89-1.00)	.04	14.9 (14.2-15.6)	1.03 (0.96-1.11)	.93	10.5 (9.9-11.1)	0.97 (0.89-1.06)	.55	6.3 (5.8-6.7)	1.24 (1.10-1.41)	.001
Age group, y															
2-4															
2019	5.8 (4.9-6.7)	1 [Reference]	NA	72.6 (70.9-74.2)	1 [Reference]	NA	13.3 (12.0-14.5)	1 [Reference]	NA	7.9 (6.9-8.9)	1 [Reference]	NA	0.8 (0.5-1.1)	1 [Reference]	NA
2020	5.1 (4.2-6.0)	0.87 (0.68-1.11)	.26	69.7 (67.9-71.4)	0.87 (0.77-0.98)	.02	14.2 (12.8-15.5)	1.08 (0.92-1.26)	.35	10.6 (9.4-11.8)	1.38 (1.14-1.66)	.001	0.8 (0.5-1.1)	1.01 (058-1.75)	.98
2021	6.9 (0.7-1.1)	1.20 (0.95-1.53)	.13	71.5 (69.5-73.4)	0.95 (0.83-1.08)	.40	12.9 (11.4-14.3)	0.96 (0.81-1.14)	.67	7.9 (6.8-9.1)	1.00 (0.81-1.23)	>.99	1.3 (0.9-1.7)	1.58 (0.94-2.64)	.08
5-11															
2019	3.2 (2.7-3.6)	1 [Reference]	NA	69.6 (68.4-70.9)	1 [Reference]	NA	13.8 (12.9-14.8)	1 [Reference]	NA	9.8 (9.0-10.6)	1 [Reference]	NA	3.7 (3.2-4.2)	1 [Reference]	NA
2020	3.4 (2.9-3.9)	1.09 (0.87-1.36)	.44	66.0 (64.6-67.3)	0.84 (0.77-0.92)	<.001	15.1 (14.1-16.1)	1.11 (0.99-1.24)	.08	11.0 (10.1-11.8)	1.14 (1.00-1.29)	.048	4.6 (4.0-5.2)	1.26 (1.04-1.53)	.02
2021	3.3 (2.8-3.9)	1.06 (0.84-1.34)	.60	65.3 (63.8-66.7)	0.82 (0.75-0.89)	<.001	15.0 (14.0-16.1)	1.10 (0.98-1.24)	.10	11.5 (10.5-12.5)	1.20 (1.05-1.36)	.007	4.9 (4.2-5.5)	1.34 (1.10-1.64)	.004
12-19															
2019	2.8 (2.3-3.2)	1 [Reference]	NA	61.5 (60.0-62.9)	1 [Reference]	NA	16.1 (15.0-17.2)	1 [Reference]	NA	12.3 (11.3-13.3)	1 [Reference]	NA	7.5 (6.8-8.3)	1 [Reference]	NA
2020	3.1 (2.6-3.6)	1.13 (0.89-1.45)	.32	61.2 (59.8-62.6)	0.99 (0.91-1.08)	.80	15.8 (14.7-16.9)	0.98 (0.87-1.10)	.69	11.5 (10.6-12.5)	0.93 (0.82-1.06)	.28	8.5 (7.7-9.4)	1.15 (0.98-1.34)	.08
2021	3.4 (2.8-3.9)	1.23 (0.96-1.58)	.10	62.0 (60.5-63.4)	1.02 (0.94-1.12)	.62	15.4 (14.3-16.5)	0.94 (0.84-1.06)	.34	10.6 (9.7-11.5)	0.85 (0.74-0.97)	.01	8.8 (8.0-9.7)	1.19 (1.02-1.40)	.03

Abbreviations: NA, not applicable; OR, odds ratio.

^a^ Compared with 2020, no significant changes in prevalence overall and across age groups were observed in 2021. Binomial logistic regression models were adjusted for age group, sex, residence (zip code groups), health insurance type, and survey weights.

 We observed the greatest increase in obesity prevalence among children aged 5 to 11 years (vs 2-4 and 12-19 years). Although healthy weight was significantly lower for this population in 2020 and 2021, their prevalence of both obesity and severe obesity was significantly higher in 2020, persisting into 2021 (11.5% [10.5%-12.5%]; *P* = .007 and 4.9% [4.2%-5.5%]; *P* = .004), compared with 2019 (9.8% [9.0%-10.6%] and 3.7% [3.2%-4.2%]). Their total unhealthy BMI prevalence (ie, overweight, obesity, and severe obesity) was also higher in 2020 (33.6% [32.7%-34.6%]) and 2021 (32.9% [32.0%-33.8%]) compared with 2019 (31.9% [31.0%-32.8%]). In addition, 2021 trends observed for these children were confirmed via longitudinal transition trend analysis, which suggested that cohort 2 experienced a significant upward BMI transition (18.1% [16.9%-19.3%]) compared with cohort 1 (14.0% [13.0%-15.0%]) (*P* < .001).

Finally, we observed that in 2020 and 2021 compared with 2019, female sex (OR, 1.28 [95% CI, 1.07-1.54]; *P* = .008 and OR, 1.32 [95% CI, 1.09-1.59]; *P* = .004), public insurance (OR, 1.24 [95% CI, 1.04-1.48]; *P* = .02 and OR, 1.36 [95% CI, 1.14-1.62]; *P* = .001), and residence in southeast Monroe County, a relatively high-income area (OR, 1.41 [95% CI, 1.10-1.80]; *P* = .006 and OR, 1.49 [95% CI, 1.17-1.91]; *P* = .001) were associated with higher odds of severe obesity.

## Discussion

The findings of this cohort study suggest that childhood obesity, especially among US children aged 5 to 11 years, was significantly higher after COVID-19 restrictions were imposed and persisted for multiple reasons^3,4,5^ after restriction removal in 2021. The sampling method used may have potentially introduced selection bias, and the narrow data coverage (1 US county) limits the generalizability of these findings. Obesity prevention efforts should focus on elementary school–aged youths to prevent high prevalence of unhealthy BMI.

## Notes

Supplement.
Data Sharing Statement
