# Supplementary material for: Evaluation of Unintended Consequences of COVID-19 Pandemic Restrictions and Obesity Prevalence Among Youths
Source: JAMA Netw Open. 2023 Jul 14;6(7):e2323596. doi: 10.1001/jamanetworkopen.2023.23596 (PMC10349338; doi:10.1001/jamanetworkopen.2023.23596)
Supplement: Supplement. — Data Sharing Statement [file jamanetwopen-e2323596-s001.pdf]

Data Sharing Statement

Lartey. Evaluation of Unintended Consequences of COVID-19 Pandemic Restrictions and Obesity Prevalence Among Youths. *JAMA Netw Open*. Published July 14, 2023. doi:10.1001/jamanetworkopen.2023.23596

Data
Data available: Yes
Data types: Deidentified participant data
How to access data: The data that support the findings of this study are available, and can be accessed and are subject to The Institutional Review Boards of both the IU Health Bloomington Hospital and the Indiana University-Bloomington approved processes. For further information and to request data, contact the senior author using dlohrman@indiana.edu.
When available: With publication

Supporting Documents
Document types: Other (please specify)
Additional Information: Raw Data used to produce all the results.
How to access documents: The data that support the findings of this study are available, and can be accessed and are subject to The Institutional Review Boards of both the IU Health Bloomington Hospital and the Indiana University-Bloomington approved processes. For further information and to request data, contact the senior author using dlohrman@indiana.edu.
When available: With publication

Additional Information
Who can access the data: Researchers whose proposed use of the data has been approved under a formal Data Use Agreement with IU Health Bloomington Hospital.
Types of analyses: For any purpose approved under a formal Data Use Agreement with IU Health Bloomington Hospital.
Mechanisms of data availability: With a signed data access agreement.
Any additional restrictions: No
